# Are the correlates of mind wandering consistent across younger and older adults?

**DOI:** 10.1007/s00426-026-02337-y

**Published:** 2026-07-20

**Authors:** Matthew S. Welhaf, Cathy Zhang, Maddie R. Valdez, Wouter Kool, Julie M. Bugg

**Affiliations:** 1 Department of Psychology, North Carolina A&T State University, 1601 E Market Street, Greensboro, NC 27410 USA; 2 Department of Psychological & Brain Sciences, Washington University in St. Louis, One Brookings Drive, CB 1125, St. Louis, MO 63130-4899 USA

## Abstract

The correlates of mind wandering are well studied in younger adult samples. However, little is known about the correlates of mind wandering among older adults. The current study took a comprehensive approach to investigating age-related differences in the correlates of mind wandering. Participants (*n* = 150 younger and *n* = 150 older adults) completed a series of attention tasks. During some of these tasks, participants were periodically probed to report on their mind-wandering experiences. Additionally, participants completed several questionnaires capturing theoretically relevant constructs such as motivation, affect, and dispositional factors. We used confirmatory factor analysis to assess correlations between our predictors and mind wandering at the construct level. We found that some factors (e.g., task-based motivation) were significantly correlated with mind wandering in both age groups, while other factors were significant correlates in one age group but not the other. Despite these apparent differences, many of the correlations did not statistically differ across the groups. We did find, however, that the association between behavioral attention lapses and mind wandering was significantly stronger in younger adults compared to older adults. A secondary aim was to replicate and extend prior work examining the mediating factors in the age-mind wandering relationship. Consistent with prior findings, we showed that motivational and affective factors only partially mediated the relationship between age and mind wandering. Our findings can help inform and refine theories of mind wandering by showing that key predictors of mind wandering are largely consistent across younger and older adults.

## Public significance statement

While we have a good understanding of which factors are associated with mind wandering in younger adults, less is known about which factors predict mind wandering in older adults. The current study provides novel evidence that the correlates of mind wandering are largely consistent across younger and older adults. Our findings can help inform and refine theories of mind wandering by showing that key predictors of mind wandering are largely consistent across younger and older adults.

## Introduction

The experience of mind wandering, or the shift of attention from external, goal-oriented, information towards internal, personally relevant information (Seli et al. 2018a, b; Smallwood and Schooler 2006), is a common occurrence in our everyday lives. We experience mind wandering roughly 30–50% of our waking hours (Banks et al., 2025; Kane et al., 2017; Kawashima et al., 2023), sometimes with detrimental consequences (for a review, see Mooneyham & Schooler, 2013). For example, mind wandering is often associated with poorer cognitive task performance (e.g., Randall et al., 2014), poorer educational outcomes (e.g., Smallwood et al., 2007; Pachai et al., 2016), increased driving errors (e.g., Traficante et al., 2025), and impaired job performance (e.g., Dane, 2018). Researchers have examined a range of factors that might predict propensity to mind wander. One such factor is aging: a consistent but surprising finding is that older adults report *fewer* instances of mind wandering than younger adults (Jordão et al., 2019; Maillet & Schacter, 2016). This pattern is unexpected because theoretical accounts of cognitive aging posit that older adults have reduced executive control ability, which should lead to increased mind wandering compared to younger adults (e.g., Braver & West, 2008; Hasher & Zacks, 1988).

Despite the growing literature on age-related differences in mind wandering, one question that has not been addressed is whether correlates of mind wandering propensity change from younger to older adulthood. Many studies have examined individual differences in mind wandering among younger adults, but there is a relatively smaller body of research examining this question in older adults. This may limit theory development and the generalizability of empirical claims. For example, a central claim made by the executive failures perspective of mind wandering (McVay & Kane, 2010) is that executive control ability predicts mind wandering. While this claim has been supported in studies testing younger adults (e.g., Banks & Welhaf, 2022; Kane et al., 2016; Marcusson-Clavertz et al., 2022; Rummel & Boywitt, 2014; Welhaf et al., 2024a), it is not clear if it holds in older adults, nor if the magnitude of the correlation is equivalent between age groups.

As another example, some theories of age-related differences in mind wandering suggest conscientiousness is a key predictor of mind wandering (in addition to other dispositional/motivational factors), with older adults generally being more conscientious and thus less apt to mind wander. However, previous research on the link between conscientiousness and mind wandering in younger adults is mixed, with some studies reporting null effects (e.g., Caron et al., 2023; Kane et al., 2017) and others finding a significant but weak association (Rummel et al., 2022). Other studies have found that conscientiousness correlates with mind wandering in older adults but not younger adults (e.g., Miller & Castel, 2025; Nicosia & Balota, 2021). Examining these potential changes in the correlates of mind wandering is therefore important for informing theory as well as for clarifying how mind-wandering propensity varies across the adult lifespan.

### Correlates of mind wandering in younger and older adults

Several studies have examined the relationship between executive control, motivational, and dispositional factors and mind wandering in younger and older adults (see Table 1 for a summary of findings). In general, there are several consistencies worth noting. First, in line with the executive failure view of mind wandering (McVay & Kane, 2010), which suggests that executive resources are required to inhibit mind wandering, executive abilities are negatively associated with mind wandering rates in both age groups (e.g., Krawietz et al., 2012; Shake et al., 2016; Robison et al., 2022; Welhaf et al., 2024a). However, many of these studies did not find significant relationships between executive control and mind wandering in older adults (potentially due to small sample sizes). Second, motivation/interest ratings were largely negatively and significantly associated with mind wandering in both age groups (e.g., Krawietz et al., 2012; Shake et al., 2016; Miller & Castel, 2025; Nicosia & Balota, 2021; Robison et al., 2022). Thus, both younger and older adults who are motivated in the task tend to mind wander less.

**Table 1:** Summary of studies reporting correlations between executive control, motivation/interest, and dispositional factors and mind wandering in younger and older adults

Construct	Study	Sample Sizes	Predictor	Mind Wandering Task	*r*_Younger_	*r*_Older_
Executive Control	Krawietz et al. (2012) Study 1	76 younger/23 older	Reading Span	Reading Task	–0.07	–0.36
	Krawietz et al. (2012) Study 2	63 younger/23 older	Operation Span	Reading Task	–0.03	–0.21
	Shake et al. (2016)	34 younger/34 older	Reading Span	Narrative Reading	–0.07	–0.23
				Expository Reading	–0.13	0.01
	Robison et al. (2022)	60 younger/62 older	Vigilance Decrement	PVT	0.32	0.25
	Welhaf et al. (2024a, b)	167 younger/160 older	Reaction Time Variability	SART	0.19	0.11
			d’	SART	–0.16	–0.22
Motivation/Interest	Krawietz et al. (2012) Study 1	76 younger/23 older	Post-Task Interest	Reading Task	–0.45	–0.41
	Krawietz et al. (2012) Study 2	63 younger/23 older	Post-Task Interest	Reading Task	–0.51	–0.18
	Shake et al. (2016)	34 younger/34 older	Pre-Task Interest	Narrative Reading	–0.60	–0.11
				Expository Reading	–0.23	–0.50
	Robison et al. (2022)	60 younger/62 older	Pre-Task Motivation	PVT	–0.58	0.05
	Nicosia and Balota (2021)	60 younger/60 older	Post-Task Motivation	SART	–0.22	–0.03
	Nicosia and Balota (2021)	60 younger/60 older	Post-Task Interest	SART	–0.34	–0.35
	Miller and Castel (2025)	108 younger/108 older	Post-Task Motivation	Word List Learning	–0.50	–0.51
Dispositional	Nicosia & Balota (2021)	60 younger/60 older	Conscientiousness	SART		
	Miller & Castel (2025)	108 younger/108 older	Negative Affect	Word List Learning	–0.06	–0.34
			Openness		0.32	0.29
			Conscientiousness		–0.26	–0.16
			Extraversion		–0.08	–0.23
			Agreeableness		–0.06	–0.17
			Neuroticism		–0.25	–0.17
			ADHD		–0.07	0.27
					–0.03	0.28

*PVT* Psychomotor Vigilance Task, *SART* Sustained Attention to Response Task

On the other hand, the few studies that report correlations between dispositional factors and mind wandering (Miller & Castel, 2025; Nicosia & Balota, 2021) show some similarities but also clear differences between the age groups. Specifically, greater negative affect is associated with more mind wandering in both younger and older adults. However, there appear to be some factors that are more strongly correlated with mind wandering in younger adults: those who are lower in levels of openness to new experiences and are less agreeable reported more mind wandering (Miller & Castel, 2025). Conversely, some correlations appear to be stronger in older adults: those who reported higher levels of conscientiousness reported less mind wandering, and those who were more neurotic and reported more ADHD symptoms reported greater mind wandering (Miller & Castel, 2025).

While these studies provide some insight into consistencies in the correlates of mind wandering between younger and older adults, there are a few limitations. First, given that most studies focus on testing mean differences in mind wandering rates between younger and older adults, many of these studies are likely underpowered for correlational analyses. Indeed, only two studies report sample sizes over 100 (i.e., Miller & Castel, 2025; Welhaf et al., 2024b). Thus, many of these correlations may be unreliable or unstable. Additionally, these studies did not test for differences in the magnitude of correlations between the age groups. As such, it’s not clear if these correlations would differ between younger and older adults. Finally, the reported studies (and much of the research on age-related differences in mind wandering) assess mind wandering in a single task (e.g., a reading comprehension task or a sustained attention task), which introduces task-specific measurement error. This may mean that the previously reported correlations are specific to the task in which mind wandering was assessed and not about general mind wandering propensity.

### Key mediators of the age-related reduction in mind wandering

Perhaps the most frequently asked question regarding age-related differences in mind wandering is why older adults report less of it. However, the evidence regarding which factors mediate this effect is mixed. For example, studies examining the potential mediating role of executive control processes have yielded mixed results, with two studies showing no mediating effect (Moran et al., 2021; Shake et al., 2016) but a more well-powered study (Borella et al., 2022) showing a significant mediation through measures of attention and inhibitory control processes.

There is more consistent evidence that non-cognitive factors such as motivation and interest ratings (Frank et al., 2015; Nicosia & Balota, 2021; Robison et al., 2022; Seli et al., 2021), certain personality traits (i.e., conscientiousness; Jackson & Balota, 2013; Miller & Castel, 2025; Nicosia & Balota, 2021; but see Jackson et al., 2013; Maillet & Rajah, 2016), and affective factors (Borella et al., 2022; Frank et al., 2015; Miller & Castel, 2025; Moran et al., 2021) can explain age-related differences in mind wandering. That is, because older adults report being more motivated to engage in the task they are completing, are more conscientious, and are likely to have more positive moods, their minds wander less than those of younger adults.

While these previous studies suggest that motivational, dispositional, and to a lesser degree cognitive factors can explain some variance in the age-related reduction in mind wandering, few of them have taken a more comprehensive approach, comparing which mediators best explain age-related reductions in mind wandering. To our knowledge, only two studies have simultaneously assessed motivational, dispositional, and cognitive mediators to better understand age-related differences in mind wandering. Moran et al. (2021) reported that older adults’ lower levels of anxiety and greater levels of task engagement (i.e., motivation) fully mediated the relationship between age and rates of unintentional mind wandering (i.e., mind wandering that occurred spontaneously despite participants trying to remain focused on the task; see Seli et al., 2016) and did so beyond cognitive factors. Similarly, Borella et al. (2022) reported a full mediation through attention and inhibition abilities and emotional well-being beyond other factors like working memory performance, dispositional mindfulness, and anxiety levels. One limitation of these studies though, is that they have only assessed mind wandering in a single task, which reduces the generalizability of their findings.

### The current study

The present study took a multifaceted approach to better understand age-related differences in mind wandering. Our first goal was to examine whether the correlates of mind wandering change between younger and older adults. We assessed several theoretically relevant constructs, including behavioral lapses as an indicator of executive control, state motivation, state affect, and dispositional factors (including personality, everyday cognitive failures, and self-reported ADHD symptoms). We also took a novel approach in the context of age-related studies of mind wandering and measured mind wandering propensity in four tasks to better capture the construct of mind wandering. In doing so, we aimed to minimize any task-specific influences that may have driven prior findings (e.g., for similar approaches see, Kane et al. 2016; McVay and Kane 2012b; Rummel et al. 2022; Unsworth et al. 2021). Additionally, our study provides a novel, well-powered, test of potential differences in the correlates of mind wandering between younger and older adults. We had the following hypotheses regarding potential differences in the magnitude of correlations between the age groups:

1. The correlations between behavioral lapses and mind wandering and motivation and mind wandering would be stronger in younger adults (e.g., Krawietz et al., 2012; Nicosia & Balota, 2021; Robison et al., 2022; Shake et al., 2016).
2. The correlation between conscientiousness and mind wandering would be stronger in older adults, consistent with previous work (Miller & Castel, 2025; Nicosia & Balota, 2021).

Given the lack of evidence regarding the correlation between mind wandering and other dispositional factors, we had no strong hypotheses for other factors (e.g., ADHD, cognitive failures, negative affect, and other facets of personality). Thus, the goal here was to conceptually replicate the secondary findings reported by Miller and Castel (2025).

Our second goal was to examine potential mediators of age-related differences in mind wandering in a more comprehensive and robust manner. Only a few studies have taken a comprehensive approach to address this question by examining cognitive, motivational, and dispositional factors as potential mediators. As others have suggested (e.g., Miller & Castel, 2025), such a narrow focus on potential mediators is problematic for the literature on age-related differences in mind wandering because it does not allow for a true test of which factors might explain why older adults report less mind wandering. We had the following hypotheses regarding potential mediators:

1. Although few studies have examined the role of executive resources in explaining age-related reductions in mind wandering, we hypothesized that older adults would experience fewer behavioral lapses (indicating better executive control ability), and this would partially explain why they report fewer instances of mind wandering, consistent with Borella and colleagues (2022).
2. In line with previous work, we hypothesized that task-specific motivation and dispositional factors, specifically negative affect and conscientiousness, would each predict age-related reductions in mind wandering. That is, older adults’ higher motivation, better mood, and higher levels of conscientiousness would each partially explain why they report fewer instances of mind wandering.

## Methods

### Transparency and openness

We report how we determined our sample size and describe all data exclusions, manipulations, and all measures used in the study (Simmons et al., 2012). We did not preregister this study. Data and analysis files for reproducing all results can be found on OSF (https://osf.io/9v5u3/).

### Participants

A total of 307 participants consented to participate (Old *n* = 157; Young *n* = 150). Younger adults were recruited from the undergraduate research pool at Washington University in St. Louis and completed the study for partial course credit. Older adults were recruited using Washington University’s Volunteers for Health program. Older adults were compensated $40 for completing the two-hour study. Our stopping rule for data collection was to collect at least 150 participants in each age group (with data collection continuing until the end of the semester, where this number was achieved). Older adult data collection began in Summer 2024, while younger adult data collection began during the Fall 2024 semester. All data collection finished at the end of the Fall 2024 academic semester.

We determined the sample size using multiple criteria. First, an a priori power analysis using G*Power (Faul et al., 2007) indicated that with a sample size of 150, we would have sufficient power to detect correlations as low as *r* =.23 with 80% power, *r* =.26 with 90% power, and *r* =.34 with 99% power (for all, α = 0.05). Thus, within each age group we would be sufficiently powered to detect traditional medium sized effects (Gignac & Szodorai, 2016). Second, because we were interested in examining group differences in correlations, a sensitivity analysis for comparing correlations of two independent Pearson correlations with a sample size of 150 in each group and 80% power (α = 0.05) would allow us to detect medium-sized differences in the correlation (ρ ≈ 0.33).

### Tasks and measures

Tasks and measures for the current study were adapted from several recent studies of mind wandering, attention lapses, and perceptual decision-making (Kane et al., 2021; Unsworth et al., 2021; Welhaf et al., 2025b; Zhang & Kool, 2026). Because of this, the methods and descriptions of the tasks will be similar to the previously published articles for consistency.

#### Attention lapses

##### Choice RT (adapted from Unsworth et al., 2012)

Participants responded as quickly (and as accurately) as possible to the appearance of a “+” in one of four underlined locations across the center of the screen. The cross appeared randomly (after a random time interval 300–550 ms in 50-ms intervals) in one of the four locations, with the exception that the stimulus could not appear in the same location on consecutive trials. Participants responded to the correct location by pressing one of four buttons on the keyboard (F, G, H, J), corresponding to the four possible locations. The task began with 15 practice trials followed by 210 experimental trials. The main dependent variable was the tau parameter from an ex-Gaussian model (for correct trials only). Tau captures the mean and SD of the exponential component of an individual’s RT distribution and has been proposed to reflect (at least partially) longer-than-average RTs.

##### Semantic sustained attention to response task (SART; McVay and Kane 2012a, b)

This go/no-go task required participants to press the space bar for words from one category (animals; 89% of trials) and to withhold responses to another category (vegetables; 11% of trials). Participants practiced the task for 10 trials by pressing the space bar whenever they saw a boy’s name and withholding responses to girls’ names. Following 10 unanalyzed buffer trials, participants completed 480 trials across 4 seamless blocks, each comprised of 3 seamless mini-blocks. Each mini-block presented 40 trials, with 36 “go” (non-target) trials and 4 “no-go” (target) trials, presented in a different random order for each participant. Dependent measures included intrasubject standard deviation (SD) of RTs to “go” (animal) trials and *d*’ (a measure of signal detection accuracy).

##### Arrow flanker

Participants reported the direction of an arrow at fixation (“<” vs. “>”) flanked by four distractors. After a 500 ms blank screen, a fixation cross (“+”) appeared for 350 ms, followed by the stimulus array, which presented either neutral flankers (“•”; 48 trials), congruent flankers pointing the same direction as the target (48 trials), incongruent flankers pointing the opposite direction as the target (48 trials), or incongruent flankers pointing upward (48 trials), until response (for a total of 192 trials across two seamless blocks of 96). Subjects pressed the “z” key (labeled with an “L” sticker) for left-pointing targets and the “/” key (labeled with an “R” sticker) for right-pointing targets. We used this task only to measure mind wandering in a standalone task, so no performance measures were calculated. Details on thought probes are discussed below.

##### Psychomotor vigilance task (PVT; Dinges & Powell, 1985)

Each trial began with a set of blue zeros (“00.000”) in the center of a white screen. After an unpredictable interval from 1 to 10 s (in 1000 ms increments), the zeros began counting upward in milliseconds. Participants were instructed to stop the counter by pressing the spacebar as quickly as possible, after which the zeros turned red to provide RT feedback. If the spacebar was mistakenly pressed before the timer started (resulting in a false alarm) that trial was repeated at the end of the task.

Participants completed two seamless blocks of 45 trials, for a total of 90 trials. In each block, participants completed four trials at each interstimulus interval (ISI). Additionally, five trials per block served as ‘yoked’ thought-probe trials. In block 1, these trials followed ISIs of 2, 4, 6, 8, and 10 s, while in block 2, they followed ISIs of 1, 3, 5, 7, and 9 s. These yoked trials presented participants with the blue zeros during the waiting period that were replaced by a thought probe (described below) rather than counting upward. The dependent measure was the mean RT of the slowest 20% trials (e.g., Unsworth & Robison, 2016; Welhaf & Kane, 2024; Welhaf et al., 2025b).

##### Perceptual decision making*** (***Zhang & Kool, 2026)

Each trial started with a fixation cross in the center of the screen. Participants then saw a random dot kinematogram (RDK) stimulus, where a total of 200 dots moved within a round aperture. A proportion of the dots moved together towards a coherent direction, while the remaining dots moved in random directions. We varied the proportion of dots moving in the coherent direction, referred to as “coherence level”, across trials. On each trial, this proportion was randomly selected from a uniform distribution between 10% and 50%. Participants were instructed to indicate whether the coherent dots were moving either left or right with one of two possible keyboard responses (Q and P).

To model the repetitive response requirements of the SART, 90% of the trials had the same coherent direction and therefore elicited the same correct response. This ‘dominant’ direction was randomly chosen for each participant and stayed the same throughout the entire task. The inter-trial interval was 1.5 s. The response deadline was 4 s. With 510 trials in total, this task lasted approximately 30 min. As with the Arrow Flanker, we did not calculate a performance variable for the Perceptual Decision Making task and only used it as a standalone assessment of mind wandering.

#### Thought probes

Participants responded to thought probes in four tasks: SART (27 probes), Arrow Flanker (20 probes), PVT (10 probes), and the Perceptual Decision Making (17 probes). We presented two types of thought probes across tasks. In the SART, Flanker, and PVT, we asked participants to classify their immediately preceding thoughts into one of eight thought content categories (e.g., Kane et al. 2016, 2021; McVay and Kane 2009, 2012a; Welhaf et al. 2022). The response options were: (1) “The task” (thoughts about the stimuli or responses); (2) “Task experience/performance” (thoughts about one’s task performance); (3) “Everyday things” (thoughts about normal life concerns, goals, and activities); (4) “Current state of being” (thoughts about one’s physical, cognitive, or emotional state); (5) “Personal worries” (thoughts about current worries); (6) “Daydreams” (fantastical, unrealistic thoughts); (7) “External environment” (thoughts about task-unrelated things or events in the testing room); (8) “Other.” Only the phrases in quotes above appeared on the probe screens. Mind wandering rates for these tasks were operationalized as the proportion of probes with responses 3–8.

In the perceptual decision-making task, rather than providing participants with categorical response options for their thoughts, probes asked participants to rate the focus of their attention on a sliding scale from 0 (completely on unrelated thoughts) to 100 (completely on the task). We treated this attention rating as a continuous measure of participants’ current focus during the task.

### Task-specific measures

In four tasks (Choice RT, SART, Arrow Flanker, and PVT), participants answered four questions about conative factors, including their current motivation to perform well, how interested they were in the task, how alert they felt, and how difficult they felt the task would be. These four questions were presented immediately following the practice trials of the four tasks but before the experimental trials. We presented these items prior to the start of the experimental trials, as previous work has suggested that post-task metacognitive judgements might be biased by performance reactivity (see Miller & Castel, 2025; Miller & Unsworth, 2021; Miller & Unsworth, 2025). Each item was rated via a button press on a 1–6 Likert scale with higher scores reflecting greater levels of the construct. Due to multicollinearity across these task-specific questions, we focus on the motivation items, given their relevance in prior studies of age-related differences in mind wandering.

### Dispositional measures

Following completion of the Arrow Flanker, participants answered the following questionnaires (described in order). For all questionnaires, items were rated via mouse click on a 1–5 Likert scale, with points labeled, *Not at all*,* A little bit*,* Somewhat*,* Very much*,* Extremely* (or participants could press a separate box to skip the item).

#### Childhood AD/HD rating scale IV–self-report version (ADHD; DuPaul et al., 1998)

Subjects completed 18 total items asking about childhood symptoms of inattentiveness (e.g., making careless mistakes) and hyperactivity (e.g., talking excessively). Separate inattentiveness and hyperactivity scores from the scale were created from the respective items (9 for each). Higher scores reflected more experiences of these symptoms.

#### Cognitive failures questionnaire–memory & attention lapses (CFQ–MAL; McVay & Kane, 2009)

Items asked about failures, such as forgetting things at home and leaving a step out of a task (e.g., “*I’m often unable to find something that I put away only a couple of days ago*”). There were seven regularly scored items and five reverse-scored items. Higher scores reflected greater everyday failures.

#### Positive and negative affect schedule (PANAS; Watson et al., 1988)

Subjects completed 20 items assessing their current positive and negative affect. Separate scores were created for positive and negative affect, with higher scores on each scale reflecting higher levels of affect.

#### State anxiety inventory (STAI; Spielberger et al., 1983)

Subjects completed 20 items about their current feelings and experiences (e.g., “*right now*,* I feel…*”). State anxiety was computed by summing items in each subscale, with higher scores indicating greater anxiety.

#### Big five inventory (BFI;*** (***John et al., 2008)

Subjects completed the BFI, which consists of 10 items measuring openness, 9 items measuring conscientiousness, 8 items measuring extraversion, 9 items measuring agreeableness, and 8 items measuring neuroticism.

### Assessment of attentive responding

We adopted items from the attentive responding scale (Maniaci & Rogge, 2014) to capture cases where participants were mindlessly responding to items to quickly complete the questionnaires. Specifically, we used three “infrequency” items (e.g., *“I don’t like getting speeding tickets”)* and two direct attention assessments (e.g., *“I will show I am paying attention by selecting Strongly Agree”*). We used the same scoring procedure detailed in Kane and colleagues (2021) to flag participants for inattentive responding. Specifically, for the three infrequency items, a score of 0 was given if participants selected a response on the appropriate side of neutral, a score of 1 was given if participants selected the neutral answer (i.e., “*Neither agree or disagree*”), and a score of 2 was given if participants selected a response on the inappropriate side of neutral. The two direct attention items were scored as 0 (if participants selected the correct answer), but as 1–4 for a wrong response. From these scores, we created a 5-item attentive response score. We removed participants who scored > 6 on this attentive response composite.

### RT cleaning procedures

For all tasks with RT-based dependent measures (i.e., Choice RT, SART, and PVT), we examined individual participants’ RT distributions for outliers, following identical procedures to previous work (e.g., Welhaf & Kane, 2024; Welhaf et al., 2025b, c). Specifically, for each participant and each task, we first removed RTs for error, post-error, and post-thought-probe trials. Next, we removed RTs that were anticipations (< 200 ms). Finally, from the remaining trials, we calculated a participant-specific outlier cutoff equal to their Median RT + 3*Interquartile Range. RTs larger than this value were replaced with this value.

### Data exclusions and outlier processing

We had several case-wise exclusion criteria. First, we excluded case-wise data from older adults who showed signs of possible cognitive impairment based on the Short Blessed Test (i.e., a score ≥ 5; *n* = 6). Additionally, we excluded one younger adult participant as they only contributed data to one task (the PVT) following the task-wise exclusions below. We additionally excluded one subject who reported their age as 17 (students under 18 can complete studies for credit, but their data cannot be used by the researchers per our IRB protocol). Finally, we excluded three participants (*N* = 2 older adults, *N* = 1 younger adult) for having scores > 6 on the inattentive response screener.

We implemented several task-wise exclusions that have been used in prior studies (e.g., Robison et al., 2024; Robison & Nguyen, 2023; Welhaf & Kane, 2024; Welhaf et al., 2025b). We excluded all SART data from 5 participants whose “go” accuracy was below 70%, suggesting they did not understand the task instructions. We excluded all PVT data from 7 participants who had 10 or more RTs removed (e.g., as anticipations or extreme outliers) or whose mean RT of their slowest 20% of trials was > 1000 ms. Finally, we removed data from the CRT tau measure for one younger adult and three older adults because they were deemed outliers (outside the age group Median ± 3*IQR).

### Procedure

Participants individually completed the above-described five cognitive tasks and questionnaire measures in a 2-hr session after first providing informed consent. Task order was fixed for all participants and proceeded as follows: Short Blessed Test (older adults only), Trail Making Parts A and B, Shipley Vocabulary, Demographics, Choice RT, SART, Arrow Flanker, Questionnaire Battery, and PVT. An experimenter remained present throughout the session and read aloud all task instructions. The protocol for this study was reviewed and approved by the Institutional Review Board at Washington University (Study Title: “*Controlling Attention and Memory*”, Protocol Number: #202301108).

### Analysis plan

Our first question focused on testing the differences in the magnitude of the correlations with mind wandering between younger and older adults. We first conducted confirmatory factor analyses (CFA) within each age group with full information maximum likelihood for model estimation to account for missing data. We report several fit statistics: $\:{\chi\:}^{2}$ (and associated degrees of freedom and p-value of the model); $\:{\chi\:}^{2}$/df, where values < 3 indicate adequate fit; Comparative Fit Index (CFI) and Tucker-Lewis Index (TLI), with values > 0.90 suggesting adequate fit; Root Mean Square Error of Approximation (RMSEA) and its associated 90% Confidence Interval [90% CI], with values < 0.10 indicating adequate fit; Standardized Root Mean Square Residual (SRMR), with values < 0.08 indicating adequate fit (Hu & Bentler, 1999). After estimating the correlations from the latent variable model, we compared the strength of the correlations with the mind wandering factor between the age groups using Fisher’s *r*-to-z test of independent correlations using the *cocor* package (Diedenhofen & Musch, 2015).

Our second question addressed the potential mediating role of the variable of interest in explaining age-related decreases in mind wandering. We conducted a mediation analysis in which age group (coded 0 = young adults and 1 for older adults) had a direct effect on the TUT rate factor and indirect effects through lapse factor, motivation factor, mood factor, ADHD ratings, and the five personality trait measures. These mediators were allowed to correlate with each other. Latent variable models and mediation analyses were run using *lavaan* (Rosseel, 2012).

## Results

### Final sample demographics

Following all exclusions, the final sample consisted of 288 participants (145 older adults and 143 younger adults). This sample size is large enough to provide stable estimates of correlations (Schönbrodt & Perugini, 2013) and is consistent with recent studies of age-related differences in mind wandering (e.g., Miller & Castel, 2025; Welhaf et al., 2024b). We had sufficient power to detect medium-to-large effects within each age group (*r*s between 0.20 and 0.30) based on prototypical individual differences effect sizes (Gignac & Szodorai, 2016).

Participant demographics are displayed in Table 2. Gender frequencies were similar in both groups ($\:{\chi\:}^{2}$(1) = 0.043, *p* =.835). Older adults were more educated by about three years, *t*(228.7) = 12.11, *p* <.001. Both groups were primarily White, but this was especially true of the older adults. Both groups also self-reported their current health to be “Good” to “Excellent”.

**Table 2:** Demographic data by Group

	Younger Adults	Older Adults
Age	19.44 (1.18)	70.08 (6.36)
Gender (*n* M/F)	47/95	45/99
Years of Education	13.60 (1.43)	16.50 (2.49)
Ethnicity (%)		
Asian	27.3	0.0
Black	7.0	10.3
Hispanic	2.1	1.4
White	49.7	86.2
Multi	13.3	1.4
Handedness (*n* L/R/Both)	20/122/0	12/130/2
Current Health (*n*)		
Poor	0	1
Fair	6	6
O.K.	13	13
Good	70	87
Excellent	53	35
SBT (*n*)		
0		101
1		2
2		29
3		2
4		10
Trail A (sec)	28.18 (11.89)	34.59 (12.67)
Trail A Errors	0.04 (0.19)	0.09 (0.31)
Trail B (sec)	67.65 (41.52)	74.19 (31.98)
Trail B Errors	0.31 (0.82)	0.52 (1.09)
Vocabulary	28.68 (4.05)	32.21 (4.49)

Means (SD) presented for continuous variables. Ns or percentages presented for categorical variables. Missing data for one older adult and one younger adult. *SBT* Short Blessed Test

In terms of neuropsychological performance, as expected, older adults were slower than younger adults on Trail Making A, *t*(282.77) = 4.41, *p* <.001. However, the two groups did not differ on Trail Making B performance, *t*(263.02) = 1.49, *p* =.138. Finally, older adults exhibited higher vocabulary performance, *t*(282.38) = 6.98, *p* <.001.

### Age differences among variables of interest

Table 3 provides the descriptive statistics for each group. Consistent with previous research, older adults reported fewer TUTs in each of the three probed tasks. In terms of performance measures, there were some differences between the age groups. Compared to younger adults, older adults demonstrated more RT variability in the Choice RT (higher Tau), but lower SART RT variability and had a shorter tail of their RT distribution in the PVT (i.e., lower RTs in the slowest 20% bin). Further, older and younger adults did not differ in their SART d’. These findings are largely consistent with recent empirical and meta-analytic findings showing that younger and older adults generally perform at similar levels on measures of sustained attention (e.g., Robison et al., 2022; Vallesi et al., 2021).

**Table 3:** Descriptive Statistics by Group

	Younger Adults		Older Adults			
	Mean (SD)	*N*	Mean (SD)	*N*	Test Statistic	Cohen’s d [95% CI]
1) CRT Tau	76.02 (33.15)	142	130.99 (59.34)	142	*t*(221.19) = 9.64***	1.14 [0.89, 1.39]
2) SART RTsd	131.29 (51.60)	139	120.58 (32.43)	144	*t*(230.90) = − 2.08*	–0.25 [–0.48, − 0.02]
3) SART d’	2.24 (0.87)	139	2.26 (0.84)	144	*t*(279.65) = 0.25	0.03 [–0.20,0.26]
4) PVT Slowest 20%	537.51 (117.02)	139	507.58 (93.90)	137	*t*(263.22) = − 2.34*	–0.28 [–0.52, − 0.04]
5) SART Mind Wandering	0.40 (0.22)	139	0.13 (0.14)	144	*t*(237.24) = − 12.42***	–1.49 [–1.75, − 1.22]
6) Arrow Flanker Mind Wandering	0.44 (0.27)	143	0.16 (0.18)	145	*t*(250.59) = − 10.52***	–1.24 [–1.49, − 0.99]
7) PVT Mind Wandering	0.50 (0.28)	139	0.31 (0.25)	137	*t*(272.51) = − 5.97***	–0.72 [–0.96, − 0.47]
8) Dots Attention Rating	48.16 (19.23)	139	72.23 (20.46)	115	*t*(236.91) = 9.59***	1.22 [0.95, 1.48]
9) CRT Motivation	4.81 (0.90)	139	5.67 (0.62)	145	*t*(252.08) = 9.36***	1.11 [0.86, 1.35]
10) SART Motivation	4.07 (1.09)	139	5.27 (1.09)	144	*t*(280.46) = 9.25***	1.10 [0.85, 1.35]
11) Flanker Motivation	3.83 (1.26)	143	5.22 (1.00)	145	*t*(270.29) = 10.39***	1.23 [0.97, 1.48]
12) PVT Motivation	4.06 (1.20)	143	5.30 (1.05)	145	*t*(239.83) = 9.16***	1.10 [0.85, 1.35]
13) PANAS Positive Affect	28.69 (5.99)	143	34.00 (6.70)	145	*t*(283.24) = 7.09***	0.83 [0.59, 1.08]
14) PANAS Negative Affect	22.78 (6.93)	143	14.24 (4.48)	145	*t*(242.70) = − 12.39***	–1.46 [–1.72, − 1.20]
15) STAI – State Anxiety	49.88 (9.54)	143	37.21 (8.69)	145	*t*(282.77) = − 11.78***	–1.39 [–1.65, − 1.13]
16) ADHD Inattention	21.38 (6.64)	143	17.31 (6.26)	145	*t*(284.51) = − 5.35***	–0.63 [–0.87, − 0.39]
17) ADHD Hyperactive	20.78 (6.19)	143	17.31 (6.26)	145	*t*(285.84) = − 5.42**	–0.64 [–0.87, − 0.40]
18) Cognitive Failures	35.87 (8.35)	143	31.71 (7.60)	145	*t*(282.71) = − 4.42***	–0.52 [–0.76, − 0.29]
19) Openness	34.99 (5.49)	143	36.89 (6.37)	145	*t*(280.97) = 2.72**	0.32 [0.09, 0.55]
20) Conscientiousness	31.59 (5.31)	143	35.58 (5.22)	145	*t*(285.73) = 6.42***	0.76 [0.52, 1.00]
21) Extraversion	24.45 (6.07)	143	26.43 (6.28)	145	*t*(285.89) = 2.73**	0.32 [0.09, 0.55]
22) Agreeableness	34.69 (4.52)	143	35.66 (4.26)	145	*t*(285.99) = 1.81^	0.21 [–0.02, 0.44]
23) Neuroticism	25.69 (5.36)	143	19.73 (5.25)	145	*t*(285.67) = − 9.53***	–1.12 [–1.37, − 0.87]

^ *p* <.10; * *p* <.05; ** *p* <.01; *** *p* <.001. *SART* Sustained Attention to Response Task, *PVT* Psychomotor Vigilance Task, *CRT* Choice Reaction Time, *PANAS* Positive and Negative Affect Schedule, *ADHD* Childhood ADHD Score, *CFQ* Cognitive Failures Questionnaire, *STAI* State Anxiety Score

The motivational and dispositional differences between age groups were all in the expected direction. Older adults were more motivated during every task. Older adults also self-reported fewer ADHD symptoms and everyday cognitive failures than younger adults. Older adults also tended to report better mood (higher positive affect and lower negative affect and state anxiety). Older adults were also more open, conscientious, and extraverted and less neurotic compared to younger adults. The two groups did not significantly differ in terms of agreeableness.

### Bivariate correlations

As seen in Table 4, mind wandering scores from one task were, for the most part, strongly associated with mind wandering scores from other tasks. Specifically, among younger adults the median correlation among the mind wandering measures was *r* =.53. The same correlation was surprisingly lower, but still modest, among the older adults with a median correlation of *r* =.39. Associations among behavioral lapse indicators were more variable, but this pattern was consistent across both age groups. The median correlation among behavioral lapse measures in younger adults was *r* =.17; in older adults, this was *r* =.23. Finally, affect and motivation measures were strongly associated with measures from the same putative construct. Thus, we appeared to reliably capture the underlying constructs of interest in both age groups.

**Table 4:** Zero-order correlations for younger adults (below diagonal) and older adults (above diagonal)

Variable	1	2	3	4	5	6	7	8	9	10	11	12	13	14	15	16	17	18	19	20	21	22	23
1) CRT Tau	--	**0.32**	**−0.24**	**0.25**	0.02	0.00	0.04	−0.06	0.01	0.11	0.11	−0.02	−0.06	0.29	**0.19**	0.14	0.13	**0.19**	−0.10	−0.08	−0.01	0.04	**0.18**
2) SART RTsd	**0.22**	--	0.04	**0.20**	0.11	0.07	−0.02	−0.15	0.05	0.09	−0.07	−0.05	0.01	0.04	−0.07	0.04	0.03	0.14	0.12	−0.02	0.04	−0.02	0.01
3) SART d’	−0.11	−0.08	--	**−0.24**	0.04	0.04	0.05	0.09	**0.20**	0.11	0.13	0.15	0.01	−0.08	**−0.18**	0.03	0.05	−0.07	0.07	−0.10	**−0.17**	0.01	−0.07
4) PVT Slowest 20%	0.12	**0.28**	**−0.27**	--	**0.19**	0.11	**0.18**	**−0.19**	−0.06	−0.06	**−0.21**	**−0.28**	0.03	0.14	**0.17**	0.07	0.00	**0.19**	0.04	−0.09	−0.01	0.08	0.02
5) SART Mind Wandering	0.06	**0.27**	−0.12	**0.28**	--	**0.64**	**0.41**	**−0.34**	**−0.18**	**−0.22**	**−0.21**	**−0.22**	−0.12	**0.18**	0.14	**0.23**	0.15	0.14	0.15	−0.17	−0.01	−0.11	0.04
6) Arrow Flanker Mind Wandering	0.08	**0.17**	**−0.21**	**0.19**	**0.65**	--	**0.48**	**−0.35**	−0.03	−0.10	−0.14	**−0.18**	−0.07	**0.27**	0.14	**0.17**	0.12	0.13	0.14	−0.09	−0.01	−0.08	0.07
7) PVT Mind Wandering	0.07	0.10	**−0.17**	**0.37**	**0.51**	**0.64**	--	**−0.36**	−0.05	**−0.18**	**−0.21**	**−0.31**	−0.07	0.09	0.04	0.07	0.11	0.00	0.16	0.03	0.12	0.01	0.10
8) Dots Attention Rating	−0.05	**−0.25**	0.11	**−0.33**	**−0.50**	**−0.54**	**−0.51**	--	0.10	**0.17**	**0.24**	**0.26**	0.04	−0.16	−0.03	−0.09	−0.12	**−0.19**	−0.12	0.08	−0.02	0.05	−0.12
9) CRT Motivation	**−0.25**	−0.07	0.05	0.03	−0.05	−0.05	−0.01	**0.17**	--	**0.42**	**0.39**	**0.40**	0.07	−0.07	−0.06	−0.03	−0.04	−0.05	−0.04	0.01	−0.07	0.04	−0.03
10) SART Motivation	−0.12	0.02	−0.08	0.04	**−0.23**	**−0.24**	−0.12	**0.21**	**0.50**	--	**0.59**	**0.63**	0.11	0.04	−0.15	−0.03	−0.06	−0.10	−0.01	0.06	−0.11	0.08	−0.09
11) Flanker Motivation	−0.08	−0.07	0.15	−0.14	**−0.31**	**−0.25**	**−0.22**	**0.27**	**0.41**	**0.63**	--	**0.69**	0.15	0.04	−0.15	−0.03	−0.02	−0.10	0.08	0.05	0.04	0.11	−0.06
12) PVT Motivation	−0.12	−0.08	0.01	**−0.24**	**−0.19**	−0.15	**−0.23**	**0.23**	**0.40**	**0.54**	**0.66**	--	0.13	−0.02	**−0.22**	0.01	0.01	−0.07	0.07	0.04	−0.03	**0.21**	**−0.17**
13) PANAS Positive Affect	**−0.18**	−0.09	0.08	0.10	−0.07	−0.01	0.06	−0.08	**0.24**	**0.17**	**0.18**	0.14	--	**−0.31**	**−0.39**	**−0.26**	0.00	**−0.29**	**0.30**	**0.41**	**0.46**	**0.39**	**−0.44**
14) PANAS Negative Affect	0.15	0.06	−0.04	−0.08	0.07	0.15	0.03	−0.06	−0.08	−0.10	0.03	0.04	**−0.20**	--	**0.40**	0.15	0.05	**0.30**	−0.15	**−0.24**	**−0.28**	**−0.17**	**0.56**
15) STAI – State Anxiety	0.16	0.04	−0.24	0.03	0.16	**0.19**	0.21	−0.11	−0.04	−0.16	**−0.18**	−0.11	**−0.29**	**0.55**	--	**0.23**	0.14	**0.29**	**−0.26**	**−0.24**	**−0.19**	**−0.26**	**0.44**
16) ADHD Inattention	0.13	0.16	−0.09	−0.04	**0.17**	0.09	−0.05	−0.07	−0.14	−0.05	0.00	−0.09	−0.16	**0.29**	**0.25**	--	**0.75**	**0.37**	−0.10	**−0.47**	−0.03	−0.16	**0.19**
17) ADHD Hyperactive	−0.05	0.12	0.02	−0.05	0.14	0.07	−0.02	−0.10	0.09	0.14	**0.22**	0.13	0.04	**0.23**	0.13	**0.66**	--	**0.20**	−0.01	**−0.20**	0.18	−0.01	0.10
18) Cognitive Failures	0.10	0.01	−0.20	0.03	**0.19**	**0.17**	0.09	−0.08	−0.07	−0.06	−0.12	−0.04	**−0.23**	**0.34**	**0.38**	**0.61**	**0.37**	--	−0.06	**−0.45**	−0.03	−0.06	**0.27**
19) Openness	−0.02	−0.14	−0.03	−0.06	0.01	0.08	−0.11	0.02	0.10	0.08	0.06	0.18	**0.26**	−0.04	−0.09	−0.01	0.08	0.12	--	−0.01	**0.34**	0.15	**−0.32**
20) Conscientiousness	−0.14	−0.04	0.07	0.09	−0.02	−0.09	0.05	−0.02	0.16	**0.20**	0.12	0.06	**0.28**	**−0.34**	**−0.37**	**−0.50**	**−0.26**	**−0.56**	0.00	--	**0.26**	**0.25**	**−0.25**
21) Extraversion	0.13	0.07	0.00	0.03	0.06	**0.18**	0.04	−0.04	0.11	0.05	**0.17**	0.08	**0.41**	0.01	−0.05	0.00	0.21	−0.01	**0.39**	0.06	--	**0.44**	**−0.32**
22) Agreeableness	−0.03	−0.06	0.01	−0.09	−0.12	0.04	0.03	0.08	0.13	0.14	0.16	**0.18**	0.14	−0.19	−0.12	−0.10	−0.14	−0.07	0.07	0.12	0.15	--	**−0.42**
23) Neuroticism	**0.17**	0.00	−0.11	0.01	−0.12	−0.01	0.02	0.06	−0.13	−0.13	−0.15	−0.16	**−0.35**	**0.46**	**0.44**	0.05	−0.16	**0.23**	**−0.17**	**−0.21**	**−0.21**	**−0.19**	--

*SART* Sustained Attention to Response Task, *PVT* Psychomotor Vigilance Task, *CRT* Choice Reaction Time, *PANAS* Positive and Negative Affect Schedule, *ADHD* Childhood ADHD Score. Bolded values are significant at *p *<.05

### Correlates of mind wandering in younger and older adults

To address our primary question of possible differences in the correlates of mind wandering between younger and older adults, we ran two separate CFAs (one in each age group). Model fit was largely adequate for both younger ($\:{\chi\:}^{2}$(138) = 206.920, *p* <.001, CFI = 0.912; TLI= 0.866, RMSEA [90% CI] = 0.059 [0.042, 0.075], SRMR = 0.061) and older adults ($\:{\chi\:}^{2}$(138) = 186.617, *p* =.004, CFI = 0.936; TLI= 0.902, RMSEA [90% CI] = 0.049 [0.029, 0.066], SRMR = 0.059). Table 5 displays the factor intercorrelations for each age group, and we summarize them here. Factor loadings for the latent factors in the models can be found in Appendix A.

**Table 5:** Factor correlations for younger adults (below diagonal) and older adults (above diagonal)

Construct	1	2	3	4	5	6	7	8	9	10	11	12
1) Mind Wandering	--	0.12	**–0.32**	**–0.28**	**0.24**	**0.18**	0.16	**0.19**	–0.14	0.02	–0.11	0.11
2) Lapses	**0.46**	--	–0.17	–0.23	0.11	0.06	**0.29**	–0.02	–0.04	0.09	0.04	0.14
3) Motivation	**–0.35**	–0.19	--	0.14	–0.02	–0.02	–0.10	0.06	0.06	–0.04	**0.19**	–0.14
4) Mood	–0.20	–0.18	0.19	--	**–0.31**	–0.06	**–0.43**	**0.34**	**0.46**	**0.50**	**0.41**	**–0.73**
5) ADHD Inattention	0.11	0.20	–0.07	**–0.37**	--	**0.75**	**0.37**	–0.10	**–0.47**	0.03	**–0.17**	**0.19**
6) ADHD Hyperactivity	0.10	0.05	**0.21**	**–0.20**	**0.66**	--	**0.20**	–0.01	**–0.20**	**0.18**	–0.01	0.10
7) CFQ	**0.20**	0.16	–0.11	**–0.49**	**0.61**	**0.37**	--	–0.06	**–0.45**	–0.03	–0.06	**0.27**
8) Openness	0.02	–0.08	0.12	0.16	–0.01	0.08	0.12	--	–0.01	**0.34**	0.16	**–0.32**
9) Conscientiousness	− 0.05	–0.08	**0.18**	**0.50**	**–0.50**	**–0.26**	**–0.56**	0.00	--	**0.26**	**0.25**	**–0.25**
10) Extraversion	0.15	0.12	0.15	0.15	0.01	**0.21**	–0.01	**0.39**	0.06	--	**0.44**	**–0.32**
11) Agreeableness	–0.02	–0.11	**0.22**	**0.23**	–0.11	–0.14	–0.07	0.07	0.12	0.15	--	**–0.42**
12) Neuroticism	–0.04	0.09	**–0.20**	**–0.64**	0.05	**–0.16**	**0.23**	**–0.17**	**–0.21**	**–0.21**	**–0.19**	--

*ADHD* Childhood ADHD Score, *CFQ* Cognitive Failures Questionnaire. Bolded values are significant at *p* <.05

In younger adults, we replicated several correlations of interest from prior research. First, increases in behavioral lapses (indicative of lower executive control ability) were associated with a greater propensity to mind wander, lower motivation, and greater everyday cognitive failures. Further, lower levels of motivation, worse mood, and greater everyday cognitive failures were associated with increased mind wandering propensity. In older adults, increases in behavioral lapses were associated with worse mood and greater everyday cognitive failures. Likewise, lower motivation, worse mood, higher ADHD symptoms, and higher openness ratings were associated with greater mind wandering propensity in older adults. Collectively, then, correlates of mind-wandering rates are largely consistent across the age groups (see Table 6). The only relationship that significantly differed between age groups was between mind wandering and behavioral lapses. Specifically, younger adults who had more behavioral lapses reported significantly more mind wandering (*r* =.46), but this correlation did not reach significance for older adults (*r* =.13). A Fisher’s test of independent correlations confirmed that these two correlations differed, Z = 3.078, *p* =.002.

**Table 6:** Comparison of Mind Wandering Correlations between age groups

Variable	Young	Old	Absolute Difference	Fisher’s Z Test
Lapses	**0.46**	0.13	0.33	Z = 3.078, *p* =.002
Motivation	**–0.35**	**–0.32**	0.03	Z = − 0.284, *p* =.777
Mood	–0.20	**–0.28**	0.08	Z = 0.713, *p* =.476
ADHD Inattention	0.11	**0.24**	0.13	Z = − 1.128, *p* =.259
ADHD Hyperactivity	0.10	**0.18**	0.08	Z = − 0.686, *p* =.493
CFQ	**0.20**	0.16	0.04	Z = 0.347, *p* =.729
Openness	0.02	**0.19**	0.17	Z = − 1.447, *p* =.148
Conscientiousness	–0.05	–0.14	0.09	Z = 0.763, *p* =.445
Extraversion	0.15	0.02	0.13	Z = 1.101, *p* =.271
Agreeableness	–0.02	–0.11	0.09	Z = 0.759, *p* =.448
Neuroticism	–0.04	0.11	0.15	Z = − 1.263, *p* =.207

Bolded values are significant at *p* <.05

### Which factors mediate age-related differences in mind wandering?

To address our second question regarding the mediating effects of these different correlates, we first conducted a simple path analysis predicting the mind-wandering factor from age group without any covariates. As expected, there was a significant negative effect of age (*ß* = –0.64, SE = 0.10, [95% CI = –0.72, –0.57], *p* <.001). Age explained 41% of the variance in the latent mind wandering factor.

**Fig. 1:**
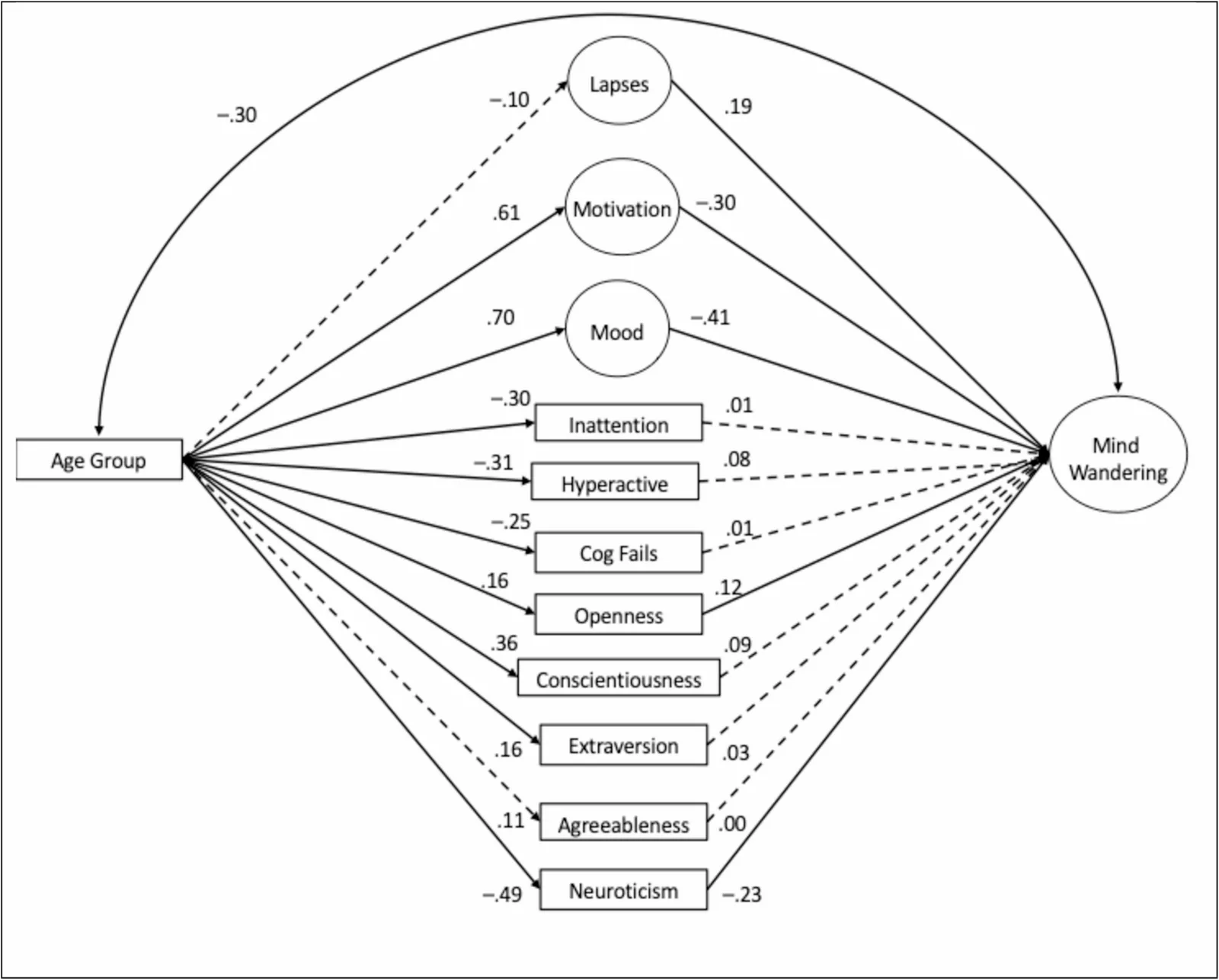
Mediation model of age-related differences in mind wandering. Solid lines are significant at *p* <.05. Dashed lines are non-significant paths

We next conducted a full mediation model that included all covariates. The resulting model is shown in Fig. 1. There were three indirect effects: motivation (*ß* = –0.17, SE = 0.04, [95% CI = –0.26, –0.09], *p* <.001), mood (*ß* = –0.33, SE = 0.12, [95% CI = –0.57, –0.09], *p* =.007), and neuroticism (*ß* = 0.13, SE = 0.05, [95% CI = 0.03, 0.22], *p* =.009). That is, older adults reported less mind wandering, in part, because they were more motivated and had a better mood. The indirect effect via neuroticism is surprising and possibly a result of high shared variance with other predictors (e.g., neuroticism-negative affect *r* = –.79). Given this, we do not consider it further. All other indirect effects were not significant (*p*s > 0.078; see Table 7). Critically, there was still a significant, albeit weaker, direct effect of Age Group on mind wandering (*ß* = –0.29, SE = 0.09, [95% CI = –0.47, –0.11], *p* =.001). Thus, motivational and affective factors appear to only partially explain why older adults report less mind wandering. In contrast, cognitive, dispositional, and neuropsychiatric factors (i.e., everyday cognitive failures; personality traits; ADHD history) did not explain age-related reductions in mind wandering.

**Table 7:** Summary of Indirect Effects for the Age-Mind Wandering Mediation

Mediator	ß	SE	95% CI	*p*
Lapses	–0.020	0.02	–0.059, 0.019	0.317
Motivation	–0.182	0.04	–0.268, –0.097	< 0.001
Mood	–0.289	0.12	–0.517, –0.061	0.013
ADHD Inattention	–0.002	0.03	–0.050, 0.046	0.949
ADHD Hyperactivity	–0.026	0.02	–0.071, 0.019	0.264
CFQ	–0.001	0.02	–0.035, 0.032	0.936
Openness	0.019	0.01	–0.002, 0.040	0.078
Conscientiousness	0.033	0.03	–0.015, 0.081	0.179
Extraversion	0.005	0.01	–0.014, 0.023	0.626
Agreeableness	0.000	0.01	–0.011, 0.011	0.968
Neuroticism	0.110	0.05	0.019, 0.202	0.018

*ADHD* Childhood ADHD Score, *CFQ* Cognitive Failures Questionnaire

### What about task-related interference?

While our primary focus was to examine age differences in the correlates of mind wandering (defined as task-unrelated thought), a form of thought that frequently increases with age is task-related interference (TRI) or internally focused thoughts related to evaluating task performance. To address age-related differences in the correlates of TRI, we conducted a complementary series of analyses using TRI rates from three tasks (SART, Arrow Flanker, and PVT; note that the thought probes presented during the perceptual decision making task did not include a response option for TRI and so it was not included in these models).

First, among younger adults, more frequent reports of TRI were significantly associated with higher task motivation (*r* =.28, *p* =.005) and better mood (*r* = –.23, *p* =.028). TRI was not associated with any other construct. Thus, younger adults who were more motivated and in a better mood reported more task-evaluative thoughts during the tasks. For older adults, TRI was not associated with any individual difference measures.

Finally, in a simple mediation model, age group significantly predicted TRI such that older adults reported more TRIs than younger adults (*ß* = 0.34, SE = 0.06, [95% CI = 0.24, 0.46], *p* <.001). Age explained 11.6% of the variance in the latent TRI factor. In a full mediation model, with all individual difference factors included as mediators, there was only one significant indirect effect through motivation (*ß* = 0.12, SE = 0.06, [95% CI = 0.01,0.23], *p* =.031). Older adults reported more TRI, in part, because they were more motivated to perform well on the tasks. Thus, not only does motivation explain in part why older adults stay focused on the task (i.e., report less mind wandering), but it also explains in part why older adults report more frequently evaluating their task performance.

## Discussion

The current study used latent variable modeling to investigate whether the associations between mind wandering and theoretically relevant predictors differ across age groups. Specifically, we examined potential age differences in the correlations between mind wandering (assessed in multiple tasks) and several theoretically relevant factors, including behavioral lapses, task-specific motivation, and dispositional factors such as negative affect, personality, and ADHD symptoms. Additionally, we aimed to add to the growing body of literature exploring possible mediators of the relationship between age and mind wandering using a more comprehensive and robust measurement approach.

### Are the correlates of mind wandering similar in younger and older adults?

The central goal of the current study was to examine whether the correlates of mind wandering differed between younger and older adults. Our results suggest that the correlations were largely similar across the age groups, which is consistent with the few (underpowered) studies previously reviewed. However, some associations showed unique patterns between the age groups.

Our hypothesis of stronger correlations between behavioral lapses and motivation with mind wandering in younger adults was partially supported. Specifically, among younger adults the association between propensity for behavioral lapses and mind wandering (*r* =.46) was not only significant, but it was significantly stronger than the correlation for older adults (*r* =.13). The finding from the younger adult sample replicates previous work which argued that the modest-to-strong correlation between behavioral lapses and mind wandering propensity might reflect a more general construct of attention consistency (Unsworth & Miller, 2021; Welhaf & Kane, 2024; Welhaf et al., 2025b). However, it is worth noting that several studies have reported null associations between task performance and reports of mind wandering in older adult samples (e.g., Jackson & Balotan 2013; Nicosia & Balota, 2021; Welhaf et al., 2024a, b; but see Robison et al., 2022). Future work will have to further address this issue by taking a more comprehensive assessment of cognitive resources (e.g., working memory capacity, attention control, processing speed).

The null correlation between attention lapses and mind wandering in older adults is somewhat surprising but could perhaps be explained by older adults recruiting different mechanisms during the attention tasks. From the executive failures perspective, mind wandering reflects a momentary lapse of executive control processes resulting in a failure of goal maintenance (e.g., McVay & Kane, 2009). However, there is another attentional process that operates to promote task performance which may be less (if at all) affected by mind wandering. This process is described as a response competition process which reflects bottom-up resolution of interference information even when task-goals are appropriately activated (i.e., Kane & Engle, 2003). Note that goal-maintenance and competition resolution are conceptually similar to the Dual Mechanisms of Cognitive Control Framework (Braver, 2012), which proposes there are two modes of cognitive control at work: proactive and reactive control. Thus, if mind wandering reflects momentary failures of goal-maintenance, then it is not surprising to see modest correlations between task errors or lapses and mind wandering propensity (as shown in the younger adult sample).

Despite older adults having well-documented deficits in goal-maintenance/proactive control processes, there is a growing body of research showing that reactive control appears relatively spared (e.g., Ball et al., 2023; Bugg, 2014; Ileri-Tayar et al., 2025). As such, older adults may be relying on reactive control processes to perform these attention tasks, and so errors and lapses are more likely a reflection of reactive control ability. If this is the case, the null correlation between lapses and mind wandering in older adults makes sense, given that these are two distinct processes. To our knowledge, only two studies have explicitly tested the reactive control-mind wandering relationship in the context of cognitive aging (Fountain-Zaragoza et al., 2016, 2018). In both cases, indices of reactive control derived from a continuous performance task were unrelated to mind wandering rates during the task. It would be fruitful to explore this dissociation in future work to better understand the association between behavioral lapses and mind wandering, especially in older adults.

Regarding motivation, we also hypothesized a stronger correlation in younger adults, possibly due to a restricted range issue. That is, older adults are much more motivated and interested in engaging in research studies and basic cognitive tasks (for reviews see Carr et al., 2022; McNeil et al., 2016; Ryan & Campbell, 2021). However, our results suggest that motivation was strongly and similarly correlated with mind wandering in both age groups, which is inconsistent with some prior work (e.g., Nicosia & Balota, 2021). Thus, although older adults were more motivated to perform each task, this did not change the overall rank-ordering of individuals between the groups.

Finally, we found that four dispositional factors showed unique associations between the age groups. First, younger adults who experienced greater everyday cognitive lapses (i.e., higher CFQ scores) were more likely to mind wander during the lab tasks (*r* =.20). However, this correlation did not reach statistical significance for older adults (*r* =.16). Second, among older adults, those who were higher in openness were surprisingly more prone to mind wandering (*r* =.20). However, this correlation was not evident in younger adults (*r* =.02). These findings related to openness add to a small body of literature showing mixed evidence between personality and mind wandering. For example, some studies report a positive association between openness and mind wandering in daily life among younger adults (e.g., Kane et al., 2017; but see Zawadski et al., 2024 for a negative finding). Studies assessing mind wandering during ongoing tasks have shown either negative (e.g., Miller & Castel, 2025) or null associations (e.g., Smeekens & Kane, 2016; Unsworth et al., 2021) between openness and mind wandering for younger adults. Further, Miller and Castel (2025) reported a nonsignificant negative correlation between openness and mind wandering in their older adult sample (*r* = −.16). One issue that is apparent in many studies investigating age related differences is that older adults who participate in research studies may be less representative of the general population and so there may be a self-selection bias which contributes to cognitive- and personality-related findings. For example, older adults may be more open to new experiences, such as engaging in scientific research. This may be less prevalent among younger adults, because participation is often required. Given the inconsistencies across the literature and the lack of research examining how openness is related to mind wandering in older adults, further replication is needed to better understand this association.

Third, among older adults, those who reported experiencing more ADHD symptoms in childhood reported more mind wandering (*r*s = 0.24 and 0.18 for Inattention and Hyperactivity facets, respectively). This finding is consistent with Miller and Castel (2025), who examined current ADHD symptoms. These correlations were not evident in younger adults, consistent with Beikmohamadi and Meier (2024: but see Meier, 2021). Finally, older adults who reported worse mood appeared to mind wander more (the correlation was just non-significant in younger adults). Despite these unique associations within each age group, it is important to note that the magnitude of these correlations did not differ between the groups. Thus, many correlates of mind wandering in younger adults appear relatively stable into older adulthood.

### Mediators of the age-mind wandering relationship

Our secondary focus was aimed at more comprehensively examining mediators of age-related changes in mind wandering. Our findings largely replicated previous findings suggesting that motivational and affective factors both partly account for decreased mind wandering in older adults compared to younger adults. That is, there were significant indirect effects through the motivation factor and the mood factor (and an unexpected indirect effect through Neuroticism). Despite these indirect effects, there was still a direct effect of age on mind wandering. Despite our similar approach to Miller and Castel (2025), we found one interesting difference in our mediational analyses. Namely, Miller and Castel found that after accounting for the key mediators, older adults demonstrated *more* mind wandering. Our study, on the other hand, found that older adults reported less mind wandering. One key difference between our studies that could contribute to these discrepancies could be how participants were recruited. Specifically, Miller and Castel collected data from younger and older adults on Prolific and tested remotely, without experimenter observation, while participants in our study were recruited locally and tested in-person. While some studies demonstrate similar experimental- and age-related effects in online settings (see Greene & Naveh-Benjamin, 2022), it is possible that there may be important differences between older adults from Prolific and those recruited in person (e.g., intrinsic vs. extrinsic reasons for engaging in research).

It is possible that other cognitive and dispositional factors that have not been assessed might also be worth considering. While previous research has focused on executive resources captured by working memory and attention control/lapses, other cognitive factors might be important mediators. For example, recent work has proposed that differences in cognitive flexibility or switching ability might explain age-related reductions in mind wandering (Wong et al., 2023). Wong and colleagues (see also Murray & Krasich, 2022) propose that mind wandering reflects instances of mental set shifting and argue that healthy older adults (compared to younger adults) have declines in shifting ability and so cannot effectively switch between periods of being on-task and mind wandering. As such, future work might consider measuring shifting ability along with behavioral lapses and working memory capacity as possible cognitive mechanisms behind age-related differences in mind wandering.

Recent work has also examined differences in neurocognitive integrity between younger and older adults (Martinon et al., 2019) as a possible reason for age-related differences in mind wandering. Martinon and colleagues found that older adults had reduced connectivity between areas of the brain that support mind wandering, such as the default mode and frontoparietal networks. Additionally, among older adults, this reduced connectivity was not associated with poorer creativity performance (which the authors used as a proxy for general cognitive ability). The authors suggest that age-related reductions in mind wandering may be related to disruptions in areas central to supporting mind wandering and not simply declines in cognitive resources. Thus, exploring the possible neural mechanisms underlying age-related differences in mind wandering could provide additional evidence for why older adults mind wander less (see also Henderson et al., 2024; Maillet et al., 2019, 2020; Maillet & Rajah, 2016).

There are also several dispositional factors that might help explain age-related differences in mind wandering that were not assessed in the current study. For example, older adults tend to report lower levels of perceived stress (e.g., Scott et al., 2013) and greater levels of mindfulness (e.g., Mahlo & Windsor, 2021) compared to younger adults, and both are related to mind wandering in younger adults. Recent work has shown that age-related differences in mind wandering are still evident after controlling for perceived stress (Welhaf et al., 2025c) and mindfulness (Borella et al., 2022; Frank et al., 2015). These factors, along with the cognitive and motivational factors previously discussed, provide additional opportunity to provide a more complete picture of why older adults report less mind wandering than younger adults.

### Further distinguishing Task-Related Interference and mind wandering

Consistent with several prior studies, older adults reported more TRIs compared to younger adults (e.g., Frank et al., 2015; Henderson et al., 2024; Jordano & Touron, 2017; McVay et al., 2013). At the individual differences level, younger adults who were more motivated and in a better mood reported fewer TRIs. The same was not true for older adults. However, consistent with the mind wandering analyses, motivation levels significantly mediated the relationship between age group and TRIs. We speculate that these results might be due in part to older adults’ error monitoring (and error minimization strategies) over the course of the task. As one example, there is a well-documented finding that older adults show larger post-error slowing compared to younger adults (see Vallesi et al., 2021 for a meta-analysis). In fact, some theories of post-error slowing suggest that this cautious slowing arises from some form of brief attentional lapse (e.g., Notebaert et al., 2009). Thus, older adults may not only be more motivated to fully focus on the task resulting in lower mind wandering rates, but they may also be more motivated to reflect on their errors during tasks and how to avoid them on subsequent trials, resulting in increased TRI rates. Future work should aim to explore which factors might also explain these age-related differences in TRI to better understand the ongoing conscious experiences of older adults.

### Limitations and constraints on generality

While the present work has many strengths, including combining more robust and comprehensive approaches to studying age-related differences in mind wandering in a larger-than-typical sample size, there are some limitations worth noting. First, our definition of mind wandering centered around task-unrelated thoughts. While task-unrelated thoughts are a common operationalization of mind wandering, other facets of mind wandering might provide unique insights into individual- and age-related differences. For example, previous work has shown that age-related differences in mind wandering might be distinct for intentional and unintentional mind wandering reports (Moran et al., 2021; Seli et al., 2017, 2021), emotionally-valenced mind wandering (e.g., Welhaf et al., 2024b, 2025c), and the temporal orientation of mind wandering (e.g., Jackson et al., 2013; Maillet & Schacter, 2016; but see Maillet et al., 2018).

Second, while one key strength of our study is the assessment of mind wandering in multiple tasks and using latent variable modeling to assess construct-level associations, it is worth noting that all of the tasks in which mind wandering was assessed were relatively demanding on attention control abilities. Although age-related differences in mind wandering are evident across different experimental contexts, there is some evidence to suggest that the age-related difference in mind wandering might be greater in more demanding tasks (Jordão et al., 2019). Future work might consider incorporating tasks with lower cognitive demands to provide a more multifaceted approach to understanding differences in the correlates of mind wandering in older adults (see Robison et al., 2020).

Third, although our sample size was larger than most studies examining age-related differences in mind wandering, we may have been underpowered to detect smaller correlations within each age group (especially among our older adults) and underpowered to detect smaller differences between the age groups. That is, we were sufficiently powered to detect medium-sized differences between the age groups, but many of the differences in correlations were small in magnitude, on average about 0.10. One possible source of this power issue might be in part due to differences in within-group variability. Specifically, older adults appeared more homogenous (i.e., reduced ranges) on some of the mind wandering and lapse measures. For example, among younger adults the range of mind wandering rates on the SART spanned from 0% to 96%, but for older adults, this ranged only from 0 to 63%. Indeed, Levene’s tests of differences in variances on the mind wandering measures indicated significant age differences on the SART and Arrow Flanker (*p*s < 0.001), but no differences on the PVT and perceptual decision making task (*p*s = 0.605 and 0.280, respectively). Likewise, there were significant age differences in variance on lapse measures of CRT tau and SART RT variability (*p* <.001), but no differences on SART d-prime and PVT Slowest 20% trials (*p*s = 0.280 and 0.054, respectively). These differences may be less problematic in the current study, given our use of latent variable models, which capture shared variance across measures, rather than relying on a single task such as the SART. We think the current results can provide a useful starting point for future work. Still, larger samples with multiple tasks (with tasks that vary in difficulty and produce more variability in mind wandering) will be necessary to better understand how correlates of mind wandering may differ between younger and older adults.

Finally, our findings are cross-sectional in nature. Thus, we do not know which factors might explain how the experience of mind wandering changes over time and declines with age within an individual. While some work has begun to address this question within older adult samples (see Welhaf et al., 2024a, 2025a), this remains an understudied line of research that should be explored to further develop theories of mind wandering and refine the implications that age has on these theories.

## Conclusions

The current study aimed to better understand age-related differences in mind wandering by testing for differences in the correlates of mind wandering between younger and older adults using a latent variable assessment of mind wandering. Our results suggest some similarities and some differences across the two groups. Regarding similarities, individuals from both age groups who were less motivated and had a worse mood were more prone to mind wander (and the magnitude of these correlations was statistically similar). On the other hand, behavioral lapses were significantly associated with mind wandering in younger adults, but this was not the case for older adults. Further, we replicated and extended recent work showing that motivational and affective factors are key mediators in the age-mind-wandering association. Our study provides evidence that while age-related differences in mind wandering are robust, it is important to take a multifaceted approach to fully understand why older adults report less mind wandering.

## Data Availability

Data and analysis files for reproducing all results can be found on OSF (https://osf.io/9v5u3/?view_only=7c690369123b4642b8a47a1a76661638).

## Appendix A

**Table 8:** Standardized Factor Loadings (With Standard Errors) for each age group

Construct and Measures	Age Group	
	Younger	Older
Lapses		
CRT Tau	0.33 (0.10)	0.59 (0.10)
SART RTsd	0.59 (0.12)	0.46 (0.11)
SART d’	–0.44 (0.12)	–0.48 (0.12)
PVT Slowest 20%	0.53 (0.10)	0.50 (0.10)
Mind Wandering		
SART Mind Wandering	0.76 (0.05)	0.78 (0.05)
Arrow Flanker Mind Wandering	0.85 (0.04)	0.79 (0.05)
PVT Mind Wandering	0.72 (0.05)	0.60 (0.07)
Dots Attention Rating	–0.66 (0.06)	–0.52 (0.09)
Motivation		
CRT Motivation	0.55 (0.07)	0.50 (0.07)
SART Motivation	0.76 (0.05)	0.74 (0.05)
Flanker Motivation	0.84 (0.04)	0.79 (0.04)
PVT Motivation	0.75 (0.05)	0.87 (0.04)
Mood		
PANAS Positive Affect	0.47 (0.09)	0.70 (0.07)
PANAS Negative Affect	–0.74 (0.06)	–0.70 (0.07)
STAI – State Anxiety	–0.72 (0.06)	–0.56 (0.07)

*SART* Sustained Attention to Response Task, *PVT* Psychomotor Vigilance Task, *CRT* Choice Reaction Time, *PANAS* Positive and Negative Affect Schedule, *STAI* State Anxiety Score

## Declarations

### Dissemination Note

The ideas and data appearing in this manuscript have not been disseminated elsewhere.
